# Adapting WHO integrated care for older people (ICOPE) models to the Korean context: Policy relevance and feasibility-a Delphi survey

**DOI:** 10.1016/j.jarlif.2025.100031

**Published:** 2025-12-31

**Authors:** Hee-Sun Kim, Chang Won Won, Yunhwan Lee

**Affiliations:** a Health Care Assessment Research Division, National Evidence-based Healthcare Collaborating Agency (NECA), Seoul 04933, South Korea; b Department of Family Medicine, College of Medicine, Kyung Hee University, Kyung Hee Medical Center, Seoul 02447, South Korea; c Department of Preventive Medicine and Public Health, Ajou University School of Medicine, Suwon, South Korea

**Keywords:** Integrated care, ICOPE, Older adults, Delphi method, Primary care, Health policy, Korea

## Abstract

**Background:**

Korea is projected to become a super-aged society by 2025, prompting the World Health Organization (WHO) to develop the Integrated Care for Older People (ICOPE) framework for person-centered, community-based care. Despite Korea’s efforts in translating ICOPE materials and launching pilot projects, evidence on their national policy alignment and feasibility for scale-up remains limited.

**Objective:**

This study assesses the policy relevance and feasibility of implementing the ICOPE framework in Korea through expert consensus and structural comparison with ongoing national and local initiatives.

**Methods:**

A three-round Delphi survey involved 31 experts in geriatrics, public health, and aging policy. Thirty-two ICOPE implementation items were evaluated across micro, *meso*, and macro levels using interquartile range (IQR) and content validity ratio (CVR). Results were compared with key Korean programs, including the Seoul Health Companion Center and Ministry of Health and Welfare’s Integrated Care Pilot Project.

**Results:**

Most ICOPE elements, particularly at the micro level (e.g., frailty screening in primary care: IQR = 0.93, CVR = 0.72), were deemed relevant and feasible. However, macro-level components like digital infrastructure and financing showed limited readiness.

**Conclusion:**

The ICOPE framework is broadly compatible with Korea’s integrated care strategies, especially at the micro level. Macro-level implementation gaps persist. This study’s findings, particularly identified consensus priorities and policy-practice gaps, aim to directly inform future policy design and scale-up strategies for integrated care in Korea, guiding efforts in governance integration, ICT infrastructure development, and indicator standardization.

## Highlights

• Korea’s aging care policy was evaluated using the WHO ICOPE framework.
• A Delphi survey identified priority items for implementing integrated elderly care.
• Strong expert consensus was reached on the feasibility and relevance of micro-level actions like frailty screening.
• System-level readiness was lower for ICT infrastructure, legal frameworks, and financing.
• Findings highlight the potential for aligning Korean policies with global care standards.

## 1 Introduction

As global aging accelerates, the World Health Organization (WHO) developed the Integrated Care for Older People (ICOPE) framework for person-centered, community-based care, emphasizing intrinsic capacity preservation and integrated service delivery across health and social sectors [1,2]. ICOPE provides a three-tiered implementation approach: micro (individual-level), *meso* (community-level), and macro (system-level) [1,5].

Korea faces one of the world's most rapid demographic transitions, projected to become a super-aged society by 2025 [[6], [7], [8], [9], [10], [11]]. In response, the Korean government initiated programs like the Community Integrated Care Pilot Project and enacted the 2024 Act on the Integrated Support for Medical and Long-term Care [11,12]. Local governments also launched municipal-level programs to address service fragmentation [13]. Despite these efforts, Korea's integrated care system remains fragmented, with limited evidence on its alignment with the ICOPE framework [1,5,7,8]. In the Korean context, studies report a wide range of frailty prevalence among older adults (e.g., from 2.5 % to 55.7 %) [14], with a recent cohort study finding physical frailty prevalence at 6.3 % [15], indicating significant care needs and underscoring the urgent necessity for structured policy evaluation and comprehensive integrated care models [8].

Globally, countries like France, Singapore, China, Spain, and Japan have implemented ICOPE in diverse ways, emphasizing varied approaches from digital tools to community-based adaptations [3,5,[16], [17], [18]]. These international variations underscore the need to identify structural barriers and enablers for ICOPE adaptation in Korea.

To address this gap, this study aims to evaluate the extent to which Korea’s integrated care policies for older adults align with the WHO ICOPE implementation framework. Using a structured Delphi method, we sought to identify expert consensus regarding the feasibility, implementation status, and alignment of existing initiatives across micro, *meso*, and macro levels. The findings are expected to inform future policy design and scale-up strategies in preparation for Korea’s transition into a super-aged society.

## 2 Methods

### 2.1 Study design

**Fig. 1:**
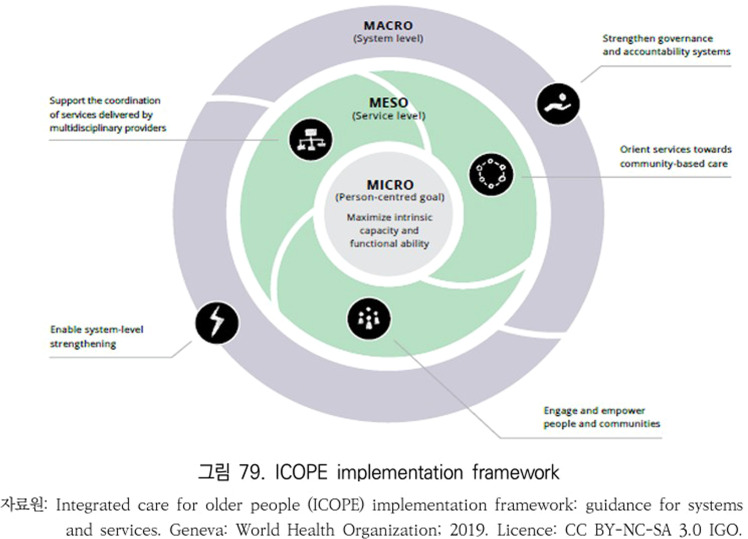
ICOPE implementation framework.

This study employed a structured Delphi method to assess the alignment of Korea’s integrated care initiatives for older adults with the WHO Integrated Care for Older People (ICOPE) implementation framework [1]. The Delphi technique is a widely accepted approach for building expert consensus in complex policy environments, particularly where empirical data are limited and stakeholder perspectives are diverse. The conceptual structure of the WHO ICOPE implementation framework is shown in Fig. 1.

### 2.2 Reference framework: WHO ICOPE

The WHO ICOPE implementation framework outlines core components and recommended actions across three levels of policy execution [1]:

• Micro level: functional ability assessment, person-centered care planning, and service personalization
• Meso level: local governance structures, multidisciplinary team-based coordination, and referral mechanisms
• Macro level: system-level integration, financing mechanisms, legal frameworks, and digital infrastructure

Survey items were developed based on these ICOPE action domains to systematically evaluate the presence, feasibility, and alignment of Korea’s current policies and practices.

### 2.3 Participants

A total of 31 national experts were recruited using purposive sampling to ensure representation across relevant sectors, including geriatrics, primary care, public health, long-term care, and health policy. 10 Participants included academic researchers, clinicians, and public officials with experience in designing or implementing elderly care policies at the national or municipal level.

### 2.4 Delphi process

The Delphi process was conducted over three rounds between July and September 2021, using data directly extracted from a previous research report [19].

• Round 1: Participants were asked to assess the degree of implementation and policy alignment for 12 key ICOPE action areas using a 5-point Likert scale (from 1 = not implemented to 5 = fully implemented). Open-ended responses were used to identify additional policy directions and practical considerations.
• Round 2: Aggregated results and anonymized group feedback were shared with participants to allow for reconsideration of initial responses.
• Round 3: Final convergence was evaluated to determine consensus priorities and identify policy–practice gaps.

### 2.5 Data analysis and ethical approval

Descriptive statistics were used to calculate means and standard deviations for each item. The level of expert consensus was assessed using the coefficient of variation (CV), with values below 0.5 interpreted as strong agreement [20]. Qualitative responses were thematically analyzed to contextualize quantitative results and derive implications for future policy development. This study was approved by the Institutional Review Board (IRB) of [NECA], approval number: [(IRB No. NECA-IRB-2021–008)].

### 2.6 Comparative policy mapping and structural analysis

The priority items identified through the Delphi process were reclassified into eight strategic policy domains of the WHO ICOPE implementation framework (Table 4). These were then compared against existing national and municipal policies, including Seoul’s Health Companion Center program and the Ministry of Health and Welfare’s Integrated Care Pilot Project [[11], [12], [13]].

The analysis examined the alignment of each Delphi-derived item with current policy efforts, focusing on the level of implementation, responsible actors, and institutional support structures.

This multidimensional mapping provided empirical insights into the contextual feasibility and system readiness for implementing ICOPE within Korea’s health policy environment.

## 3 Results

### 3.1 Results of the Delphi survey

A total of 31 experts participated in the Delphi survey. Across the 32 implementation items derived from the WHO ICOPE framework, the average interquartile range (IQR) was 0.93 and the average content validity ratio (CVR) was 0.72. Based on the predefined thresholds (IQR ≤ 0.5 and CVR ≥ 0.75), 12 items were identified as having achieved a high level of expert consensus.

This indicates a shared perspective among experts regarding the feasibility and appropriateness of implementing ICOPE-related policies. Notably, items categorized under the Micro-level—particularly those related to individualized service provision in primary care—were rated as having strong policy applicability and implementation potential. The 32 Delphi survey items were classified according to WHO ICOPE recommendations (Table 1).

**Table 1:** Delphi Survey Items Based on WHO ICOPE Recommendations and Implementation Framework.

Category	Major Domains	Subcategories (No. of Survey Items)
1. Implementation Framework for Integrated Older Adult Services (19 items)	Service Delivery	- Strengthening participation and roles of individuals and communities- Multidisciplinary support and integrated service provision (4 items)
		- Community-based care coordination and delivery (3 items)
	System-Level Governance	- Enhancing governance and accountability systems (4 items)
		- System-wide strengthening and activation (6 items)
2. Recommendations for Intrinsic Capacity-Based Integrated Care (13 items)	Decline in Intrinsic Capacity (Physical and Mental)	- Mobility decline (3), malnutrition (1), visual impairment (1), hearing loss (1), cognitive decline (1), depression (1)
	Geriatric Syndromes	- Falls (2), incontinence (4)
	Caregiver Support	- Caregiver support (1)

### 3.2 Consensus analysis by implementation level

#### 3.2.1 Micro-level

Among the 13 Micro-level items, six met the consensus criteria of IQR ≤ 0.5 and CVR ≥ 0.75. For example, the item “Recommendation of multicomponent exercise” recorded an IQR of 0.5 and a CVR of 0.77, while “Provision of cognitive stimulation programs” also showed strong consensus (IQR 0.5, CVR 0.78). These components are currently reflected in the operational guidelines of the Seoul Health Companion Center, confirming their applicability in practice.

Furthermore, the item related to caregiver support interventions demonstrated shared agreement among experts, highlighting the recognized need for policies that strengthen caregiver education and psychosocial support. National policy alignment with ICOPE implementation domains is summarized in Table 2.

**Table 2:** Analysis of National Policy Alignment with ICOPE Implementation Domains (Meso and Macro levels).

#### 3.2.2 Meso-level

The item “Efforts to screen and connect care recipients” achieved an IQR of 0.5 and a CVR of 0.81, while “Service delivery by trained community-based personnel” showed positive consensus (IQR 0.5, CVR 0.75).

However, the item “Deployment of multidisciplinary teams for individualized care planning” revealed low agreement (IQR 1.38, CVR 0.27), suggesting a lack of consensus regarding the feasibility or design of such interventions in the current policy setting.

#### 3.2.3 Macro-level

Overall, the Macro-level items showed lower consensus. For instance, “Establishing an ICT-based information-sharing system” had an IQR of 1.0 and a CVR of 0.09, indicating acknowledgment of its strategic importance but disagreement on its implementation feasibility.

In contrast, “Support for self-management using digital tools” showed relatively high convergence (IQR 0.5) and a moderate level of consensus (CVR 0.58), identifying it as a promising target for future institutionalization. Implementation levels across micro-level recommendations are detailed in Table 3.

**Table 3:** Analysis of Domestic Service Implementation Levels According to 13 ICOPE Recommendations (Micro Level).

### 3.3 Comparative analysis with national and local policy cases

A comparative analysis between the Delphi results and two key Korean policy initiatives—the Seoul Health Companion Center and the Ministry of Health and Welfare’s Integrated Care Pilot Project—revealed notable alignment, particularly at the Micro level. For example, implementation items such as the Korean Frailty Index for Primary Care (KFI-PC) [7,8,21] and individualized care planning are explicitly reflected in pilot project guidelines and operational manuals. Specifically, activities such as frailty screening using the KFI-PC and the development of personalized health plans have already been integrated into Seoul’s Health Companion Center protocols and NECA’s frailty management initiatives.

At the Meso level, some coordination mechanisms and case management practices demonstrated structural consistency. This includes the formation of multidisciplinary teams linking public health centers, primary care clinics, and pharmacies, as well as the implementation of standardized training programs for nurses within the Seoul model (Table 4a).

**Table 4a:** Comparison of WHO ICOPE Implementation Domains, Delphi Survey Items, and Korean Policy Applications.

**Implementation Level**	WHO ICOPE Strategy Domains	Delphi Survey Items	Korean Policy Applications
Micro level	Functional Decline Screening	Frailty screening based in primary care settings	Screening tools such as the KFI-PC and SPPB have been introduced in primary care settings. These indicators were applied in the study titled “Cost analysis of frailty in primary care and cost-effectiveness of integrated frailty management” [7].
	Personalized Care Planning	Implementation of individualized health management plans	Pilot operation of the Seoul Health Companion Plan; the Seoul Health Companion Center Guidelines include a dedicated section for individualized care planning [12].
Meso level	Multidisciplinary Team Formation	Coordination across local healthcare institutions	The Seoul Health Companion Team was established through linkage among public health centers, primary clinics, and community pharmacies, as outlined in the Seoul Community Integrated Care Guidelines [12]
	Community Resource Linkage	Integration of local health and social care resources	Coordinated case management between public health centers and clinics has been implemented in Seoul; multiple reports document the linkage between home-visiting nurses and clinic-based care [12]
	Workforce Training	Implementation of field-based training programs	Community nurses and primary care providers received structured training; education modules were included in NECA’s integrated frailty management research [7,12].
Macro level	ICT-Based Information Integration	Development of integrated health information systems	A pilot project for an EMR-based regional health integration platform was launched as part of Seoul’s digital healthcare initiative [22,24]
	Legal and Institutional Framework	Establishment of legal and regulatory infrastructure	The Integrated Care Support Act was enacted in 2024 to provide a legislative foundation for community-based care (MOHW press release and statute documentation) [11,13]
	Financial Infrastructure Development	Securing sustainable financing mechanisms	Seoul’s Health Companion Program initially operated with municipal funds, later linked with MOHW’s national pilot through central-local government cost matching
	User Engagement and Empowerment	Digital inclusion and self-care education	Digital literacy and self-management support services were implemented in parallel; the Ministry of Science and ICT’s digital inclusion strategy [23] and Seoul’s Senior-tailored Digital Literacy Assessment Tool [24] supported this approach.

At the Macro level, some progress has been made with the enactment of the Act on the Integrated Support for Medical and Long-Term Care in 2024 and the development of regional health integration platforms based on Electronic Medical Records (EMRs). However, significant disparities remain between regions that are part of pilot projects and those that are not. As a result, key components such as ICT integration, workforce capacity-building, and fiscal consolidation are still in the early stages of institutionalization. This gap reflects an ongoing disconnect between policy design and implementation on the ground.

These findings suggest that while the Micro- and Meso-level elements of the WHO ICOPE framework are generally aligned with Korea’s current policy strategies, the Macro-level strategies require further operationalization and specificity. Strengthening effectiveness at the Macro level will necessitate systematic incorporation of ICOPE strategic directions into the Act on the Integrated Support for Medical and Long-Term Care (enforced since March 2024). To this end, establishing interministerial governance, advancing ICT-based data-sharing platforms, and ensuring regional equity in service delivery should be prioritized in future policy development. A comparison between Delphi findings and Korean policy applications is presented in Table 4a.

## 4 Discussion

This study examined the alignment between Korea's integrated care policies for older adults and the WHO ICOPE (Integrated Care for Older People) implementation framework, based on expert consensus through a Delphi survey. The findings reveal distinct patterns across the micro, *meso*, and macro levels of implementation.

### 4.1 Micro-level alignment: strong feasibility and partial implementation

At the micro-level, substantial alignment with WHO recommendations shows robust feasibility and partial implementation in Korea's integrated care for older adults. Core interventions, such as KFI-PC screening and individualized health plans, align with WHO guidelines [7,8,21] and are evidenced by initiatives like the Seoul Senior Health Companion Center and MOHW pilot projects [[9], [10], [11], [12], [13]]. This level garnered highest expert consensus, indicating well-established implementation frameworks and promising adherence to the ICOPE framework for frailty screening and personalized care. KFI-PC is undergoing pilot testing in select primary care environments and is presently undergoing evaluation for potential widespread adoption. The national health check-up program has incorporated the Timed Up and Go (TUG) test since 2021 [25], with grip strength assessment under consideration. Pioneering initiatives from Seoul and MOHW support further micro-level expansion [11,12]. Expert consensus affirmed this care level as most practicable for immediate intervention.

### 4.2 Meso-level challenges: service coordination and workforce readiness

Meso-level integration, encompassing provider networks and inter-sectoral collaboration, shows significant gaps in Korea's healthcare system [9]. This level involves multidisciplinary care coordination and community service integration [5]. Despite community care pilots and primary care physicians' involvement in elderly care, systemic barriers persist, including fee-for-service reimbursement [25] and deeply segmented health and welfare systems [9]. Notably, recent comparative studies on community care in Asian countries also highlight these enduring challenges in Korea's fragmented medical and long-term care services [26], a concern underscored by strategic analyses of specific regional home care services facing issues like data fragmentation and workforce shortages [28]. The Seoul model, linking clinics, public health centers, and pharmacies, and EMR-based integration pilots, represent efforts to operationalize this level [22,24]. However, standardizing care protocols and professional training remains challenging [9]. The Delphi panel emphasized the need for regional coordination platforms and unified assessment tools to bridge health and welfare silos.

### 4.3 Macro-level gaps: governance, financing, and legal frameworks

At the macro level, significant policy-practice gaps persist, particularly regarding systemic infrastructure like shared information systems, cross-sector budget integration, and legal harmonization (Table 4b). Expert consensus on these macro-level items was low in our Delphi study, highlighting substantial implementation gaps at institutional and policy levels. Underdeveloped infrastructure often disconnects aspirational policy design from practical implementation [9]. Exacerbated by challenges in data fragmentation, lack of performance indicators, and limited strategic analysis of existing services in regional contexts [28], this creates specific issues. For instance, despite the Act on the Integrated Support for Medical and Long-Term Care’s comprehensive vision [11,13], its current framework offers broad guidelines not detailed operational mechanisms for inter-ministerial cooperation or integrated budgeting [9,25]. Korea’s centralized health insurance system and historical lack of specific legislation for integrated geriatric care pose structural obstacles to comprehensive ICOPE adoption [9,10]. This challenges seamless integration, as budgeting and service delivery remain siloed despite overarching policy goals [9,25]. Experts in our Delphi study emphasized the critical need for legislative amendments, incentive realignment, and institutional reforms to support robust cross-sectoral governance.

**Table 4b:** Policy Recommendations across ICOPE Implementation Levels.

Implementation Level	WHO ICOPE Strategic Domain	Delphi Survey Item (Abbreviated)	Key Findings from Delphi Consensus	Current Policy Alignment	Policy Recommend Actions
Micro	Functional decline Screening Individualized care planning	↓ Motor function ↓ Nutrition ↓ visual function ↓ Cognition/mental health Polypharmacy/falls risk Urinary incontinence Caregiver support	High consensus on feasibility of frailty screening, multicomponent exercise, and cognitive stimulation (CVR ≥ 0.72)	Strong alignment with tools like KFI-PC and Seoul Health Companion Center protocols	Scale up functional screening tools and individualized care plans at national level
Meso	Multidisciplinary team-based care Community resource linkage Workforce training	Service coordination Health/social care integration Care navigation Risk stratification Outreach/home visits Community-based referral Caregiver training Primary/community workforce capacity building	Moderate consensus on care coordination and community-based workforce (e.g., CVR = 0.75 for trained personnel)	Partial alignment with community nurse training and local team coordination models	Develop standardized multidisciplinary team protocols and expand regional integration platforms
Macro	Legal and institutional framework Financial system development User participation and empowerment ICT-based information sharing	Legislation Standardized protocols Service quality evaluation Funding models Budget integration Inclusive service design Capacity-building programs (paid/unpaid) Interoperable data systems Digital health tools	Low consensus on ICT integration, financing, and legal frameworks (CVR < 0.3 in most domains)	Limited progress, despite 2024 Integrated Care Support Act; regional disparities remain	Strengthen digital infrastructure, legal harmonization, and inter-ministerial governance for national rollout

### 4.4 Comparative insights from international models

Examining international ICOPE implementation offers insights for Korea’s integrated care model. At the micro level, countries like France and Singapore implement frailty screening and functional assessments [3,5], with high feasibility in Korea’s KFI-PC tool and Seoul’s Health Companion Center [21,28]. Korea can learn from their direct implementation approaches. Meso-level integration, key for multidisciplinary collaboration and community linkages, is common in international models [5]. France and Spain, for instance, structured integration around regional hospitals and social workers [5,17]. While Korea’s multidisciplinary team models and home visit–clinic collaboration align, integration protocols and team coordination remain underdeveloped [9]. Korea needs to learn from formalized multidisciplinary protocols and established regional collaboration platforms observed in these countries. At the macro level, robust ICT infrastructure, budget pooling, and regulatory alignment are crucial. International experiences, including Singapore and China, underscore macro-level fragmentation in digital infrastructure and financial integration [3,16], also emphasized by WHO’s 2024 report [27]. Korea faces limited EMR integration and legal infrastructure, with low expert consensus on macro readiness [9,28]. Despite the 2024 Act on the Integrated Support for Medical and Long-Term Care providing a policy foundation, regional disparities and fragmented budgeting persist [10]. Korea can draw lessons from countries that have successfully overcome these barriers through clear legislative frameworks for budget integration and national digital platforms. Comparative review with France, Japan, China, and Singapore offers specific structural and strategic lessons for Korea [5,[16], [17], [18]]. Japan’s inclusion of frailty in long-term care eligibility [18], Singapore’s strategic use of digital primary care [3], and France’s emphasis on territorial governance [5] provide particularly relevant policy design elements. These insights underline the importance of aligning digital health, local autonomy, and comprehensive geriatric competencies into Korea’s evolving integrated care models.

### 4.5 Korea's strategic response to the ICOPE framework

WHO’s 2023 and 2024 implementation reports prioritize integrated care scale-up, emphasizing digital, financial, legal, and standardization [4,27]. Table 4b summarizes Korea’s ICOPE alignment across micro, *meso*, and macro levels (Table 4b). It highlights key findings and practical recommendations to guide the expansion and institutionalization of integrated care for older adults in Korea. Korea actively pursues policy reforms to provide integrated, person-centered care for older adults, particularly through the National Community Care Program and the 2024 Act on the Integrated Support for Medical and Long-Term Care [11,13]. These initiatives link healthcare and welfare seamlessly. In clinical practice, efforts institutionalize frailty-related care pathways. For example, the Korean Society of Family Medicine issued frailty clinical guidelines in 2021 [29], and the Korean Sarcopenia Society released updated guidelines in 2023 [30]. However, due to Korea's prevailing fee-for-service reimbursement structure [25] and deeply segmented health and welfare service systems [9], it remains challenging to deliver integrated care across clinical, welfare, and preventive domains. These systemic rigidities necessitate an adaptive approach. Therefore, rather than adopting the ICOPE model in its entirety, Korea has focused on applying its core principles in a manner supporting incremental service reforms toward integration and person-centeredness. This strategic approach is reflected in institutionalized frailty screening—using adapted tools like the Timed Up and Go (TUG) test since 2021 [25] and planned grip strength assessments in 2025—within national health check-ups and long-term care eligibility screening, rather than widespread direct ICOPE tool adoption. This demonstrates a pragmatic adaptation, leveraging existing infrastructure and national priorities.

### 4.6 Contributions of this study and Korea’s role in knowledge translation

This study assessed Korea’s policy and institutional context against the WHO ICOPE framework for alignment gaps and feasible implementation strategies for integrated care. It proposed a structured diagnostic approach across micro, *meso*, and macro levels, presenting policy recommendations for enhancing service connectivity, standardization, and institutional readiness for older adult care. Our research team also advanced the ICOPE agenda in Korea through expert leadership in translating/disseminating core WHO ICOPE materials, academic discussions, and national stakeholder engagement [6]. While Delphi results have not been formally integrated into legislation like the 2024 Act on the Integrated Support for Medical and Long-Term Care [11,13], their core messages, advocating for ICOPE-based integration, were disseminated via academic and policy channels [9]. Study findings were specifically shared at conferences (e.g., Korean Geriatrics Society) and parliamentary forums [9]. Furthermore, the Korean translation of WHO’s ICOPE implementation guide was distributed to key institutions (e.g., MOHW, NHIS) to raise applicability awareness [[6], [7], [8]].

### 4.7 Study limitations and future directions

This study diagnoses Korea’s institutional readiness through the lens of the WHO ICOPE framework, offering targeted policy suggestions for micro-, meso‑, and macro-level alignment. However, several limitations should be acknowledged. First, the Delphi method reflects expert consensus but does not include the voices of older adults, caregivers, or frontline workers directly affected by integrated care delivery. Second, while the study identifies policy priorities, it does not empirically test implementation outcomes or cost-effectiveness. Third, generalizability may be constrained by the specific policy context and survey timing. Future research should incorporate qualitative and implementation science approaches to evaluate policy feasibility, acceptability, and sustainability in real-world settings. Building on this Delphi study’s insights, future work could focus on developing/evaluating specific intervention models for macro-level challenges (e.g., integrated digital platforms, pooled financing). Crucially, long-term follow-up studies needed to assess policy reforms’ actual impact on older adults’ health outcomes and quality of life. Engaging a broader range of stakeholders (including service users/families) is also essential for truly person-centered, community-responsive integrated care. Finally, translating Delphi-derived priorities into actionable policy roadmaps and evaluating their integration into national legislative/strategic planning processes remains a key area.

## IRB statement

The expert survey data used in this study were originally collected under a NECA-approved research protocol (IRB No. NECA-IRB-2021–008).

## Data availability

The datasets generated and analyzed during the current study are available from the corresponding author on reasonable request.

## Funding

This work was supported by the National Evidence-based Healthcare Collaborating Agency (Project No. NA21–006).

## Declaration of generative AI and AI-assisted technologies in the writing process

During the preparation of this work the authors used Gemini (Google's AI model) in order to improve the readability and language of the manuscript, assist in sentence restructuring, and refine overall clarity. After using this tool/service, the authors reviewed and edited the content as needed and takes full responsibility for the content of the published article.

## CRediT authorship contribution statement

**Hee-Sun Kim:** Writing – original draft, Methodology, Investigation, Formal analysis, Conceptualization. **Chang Won Won:** Writing – review & editing, Supervision. **Yunhwan Lee:** Writing – review & editing, Methodology, Funding acquisition.

## Declaration of competing interest

The authors declare that they have no known competing financial interests or personal relationships that could have appeared to influence the work reported in this paper.
